# Tunable optical Kerr effects of DNAs coupled to quantum dots

**DOI:** 10.1186/1556-276X-7-660

**Published:** 2012-11-29

**Authors:** Yang Li, Ka-Di Zhu

**Affiliations:** 1 Key Laboratory of Artificial Structures and Quantum Control (Ministry of Education), Department of Physics, Shanghai Jiao Tong University, 800 DongChuan Road, Shanghai 200240, China

**Keywords:** Kerr effects, DNA-quantum dot system, Optoelectronic material

## Abstract

The coupling between DNA molecules and quantum dots can result in impressive nonlinear optical properties. In this paper, we theoretically demonstrate the significant enhancement of Kerr coefficient of signal light using optical pump-probe technique when the pump-exciton detuning is zero, and the probe-exciton detuning is adjusted properly to the frequency of DNA vibration mode. The magnitude of optical Kerr coefficient can be tuned by modifying the intensity of the pump beam. It is shown clearly that this phenomenon cannot occur without the DNA-quantum dot coupling. The present research will lead us to know more about the anomalous nonlinear optical behaviors in the hybrid DNA-quantum dot systems, which may have potential applications in the fields such as DNA detection.

## Background

Biomaterials are now drawing more and more attention since they often present special properties which are not easily obtained from traditional inorganic or organic materials. In addition, biomaterials come from renewable resources and are usually biodegradable. Among biomaterials, researches have been interested in DNA for various reasons, such as potential applications of DNA assembly in molecular electronic devices [1], nanoscale robotics [2], and DNA-based computation [3]. One of the most interesting applications in DNA is to use DNA as a kind of optoelectronic material. Thin film of DNA-CTMA has been used successfully in various applications such as organic light emitting diodes, a cladding and host material in nonlinear optical devices, and organic field-effect transistors because of its nature of large dielectric constant and large band gap [4]. DNA-based polymers are utilized in optically pumped organic solid-state lasers [5]. A better understanding of the nonlinear optical properties of DNA materials will undoubtedly lead us to more exciting applications. So, many researches on nonlinear optical properties of DNA materials have been undertaken. Samoc et al. have studied the nonlinear refractive index and the two-photon absorption coefficient of native (sodium salt) DNA [6]. Second harmonic generation of DNA assemblies in the form of DNA-CTMA has been characterized both theoretically and experimentally by Wanapun et al. [7]. Krupka et al. investigated the third-order nonlinear optical properties of thin films of DNA-based complexes with optical third harmonic generation technique [8]. Nonlinear optical properties of different materials based on DNA are under investigation currently.

In this paper, we theoretically propose and analyze some nonlinear optical properties in a DNA-quantum dot coupling system, which have remained unexplored to date. We investigate DNA molecules coupled to the peptide quantum dot with the optical pump-probe technique. This technique has been realized by several groups [9-13], which shows the probability for experimental realization. Since photodetection technology is well developed, for instance with the assistance from quantum dot [14], we can expect to observe some properties of DNA molecules by detecting the weak probe beam. However, toxicity should always be cared about when DNA molecules are used together with nanomaterials as has been tested in [15], so a problem we need to pay attention to is that the metallic quantum dots used in biological assays are always toxic. Recently, Amdursky et al. [16,17] have shown that the peptide quantum dot is nontoxic to the environment and biological tissues. This kind of quantum dot is a good choice of new labeling materials in biological and biomedical experiments. Most recently, the coherent optical spectrum in such a quantum dot-DNA system has been studied by Li and Zhu [18].

In the system, the vibration mode of DNA molecules makes a great contribution to this coupled system so that the optical Kerr effect can be enhanced significantly. This optical Kerr effect can also be switched by adjusting the intensity of the pump beam while leaving the other parameters unchanged. In view of these novel properties, we propose a method to measure the frequency of the vibration mode of DNA molecules.

## Methods

**Figure 1:**
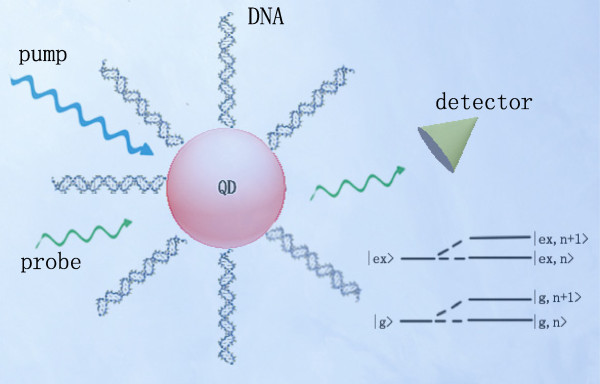
**DNA and peptide quantum dot coupling system.** A peptide QD coupled to DNA molecules in the simultaneous presence of two optical fields. The energy levels of QD when dressing the vibrational modes of DNA molecules are also shown in this figure.

To understand our system, we consider one of the large amount of DNA-quantum dots (DNA-QDs) in actual reagent as shown in Figure 1. The DNA-QD system is driven by a strong pump field and a weak probe field. A two-level system (the ground state |*g* >  and the excited state |ex > ) can be chosen as the model for quantum dot, which are dressed by the DNA vibration mode as shown in Figure 1. This two-level system can be described with the pseudo-spin 1 and the corresponding operators are *σ*_ + _, *σ*_ − _ and *σ*_*z*_. The Hamiltonian of quantum dot can be written as $H_{\mathrm{QD}}=\hbar \omega_{\mathrm{eg}}\sigma_{z}$, where *ω*_eg_ = *ω*_ex_ − *ω*_*g*_ is the exciton frequency of quantum dot.

The DNA molecules in our study are modeled as harmonic oscillators [19] and almost have no difference between them, which is not difficult to realize with biological techniques. The Hamiltonian of DNA molecules is

(1) $$\begin{array}{lcrr} H_{D}=\overset{n}{\underset{j=1}{\sum }}\left(\frac{p_{j}^{2}}{2m_{j}}+\frac{1}{2}m_{j}\omega_{j}^{2}q_{j}^{2}\right), & & & \end{array}$$

where the commutation relation $\;\;[q_{j},p_{j}]=\mathrm{i\hbar}$ is satis fied [20].

The damping of the longitudinal vibration mode of the DNA molecules is fairly small in a small volume of aqueous solution [20], though the DNA vibrational modes decay quickly. Therefore, in small volume of aqueous solution, the only vibration mode we care about is the longitudinal vibration mode. In addition, flexion of DNA molecules will result in extensions and compressions of the model, which will finally lead to the modification of the quantum dot levels through the longitudinal strain [21,22]. The Hamiltonian caused by the coupling of DNA molecules and a quantum dot has the form as follows:

(2) $$\begin{array}{lcrr} H_{\text{QD-DNA}}=\hbar \sigma_{z}\overset{n}{\underset{j=1}{\sum }}\kappa_{j}q_{j}, & & & \end{array}$$

where *κ*_*j*_ is the coupling strength between quantum dot and the *j*th DNA molecule, and the quantum dot is coupled to *n* DNA molecules. Because of the diluted aqueous solution of DNA molecules, we do not take the coupling between DNA molecules into consideration [20].

The coupling between QD and optical fields is

(3) $$\begin{array}{lcrr} H_{\mathrm{QD}-f} & = & -\mu [E_{p}\sigma_{+}\exp(-i\omega_{p}t)+E_{p}^{*}\sigma_{-}\text{exp}(i\omega_{p}t)] & \\ & & -\mu [E_{s}\sigma_{+}\exp(-i\omega_{s}t)+E_{s}^{*}\sigma_{-}\text{exp}(i\omega_{s}t)], & \end{array}$$

where *μ* is the electric dipole moment of the exciton and *E*_p_(*E*_s_) and *ω*_p_(*ω*_s_) are the amplitude and frequency of the pump-probe field, respectively.

Now we get the Hamiltonian of the QD-DNA system,

(4) $$\begin{array}{lcrr} H & = & H_{\mathrm{QD}}+H_{D}+H_{\text{QD-DNA}}+H_{\mathrm{QD}-f} & \\ & = & \hbar \omega_{\mathrm{eg}}\sigma_{z}+\overset{n}{\underset{i=1}{\sum }}\left(\frac{p_{i}^{2}}{2m_{i}}+\frac{1}{2}m_{i}\omega_{i}^{2}q_{i}^{2}\right)+\hbar \sigma_{z}\overset{n}{\underset{i=1}{\sum }}\kappa_{i}q_{i} & \\ & & -\mu [E_{p}\sigma_{+}\text{exp}(-i\omega_{p}t)+E_{p}^{*}\sigma_{-}\text{exp}(i\omega_{p}t)] & \\ & & -\mu [E_{s}\sigma_{+}\text{exp}(-i\omega_{s}t)+E_{s}^{*}\sigma_{-}\text{exp}(i\omega_{s}t)]. & \end{array}$$

In the rotating frame at *ω*_*p*_, the Hamiltonian becomes

(5) $$\begin{array}{lcrr} H & \;=\; & \hbar \Delta_{p}\sigma_{z}\;+\;\overset{n}{\underset{i=1}{\sum }}\left(\frac{p_{i}^{2}}{2m_{i}}\;+\;\frac{1}{2}m_{i}\omega_{i}^{2}q_{i}^{2}\right)-\hbar (\Omega_{p}\sigma_{+}+\Omega_{p}^{*}\sigma_{-}) & \\ & & +\hbar \vartheta \sigma_{z}-\mu [E_{s}\sigma_{+}\exp(-\mathrm{i\delta t})+E_{s}^{*}\sigma_{-}\exp(\mathrm{i\delta t})], & \end{array}$$

where *Δ*_*p*_ = *ω*_eg_−*ω*_p_, $\vartheta =\overset{n}{\underset{j=1}{\sum }}\kappa_{j}q_{j}$, $\Omega_{p}=\mu E_{p}/\hbar$ is the Rabi frequency and *δ* = *ω*_s_ − *ω*_c_is the probe-pump detuning.

With this Hamiltonian, we can obtain the equations of motion for *σ*_*z*_, *σ*_−_, and *ϑ* via Heisenberg equation and introduce some damping parameters such as *Γ*_1_, *Γ*_2_ and *τ*_D_[23]. *Γ*_1_ is the exciton relaxation rate and *Γ*_2_ is the dephasing rate. *τ*_D_ is the vibrational lifetime of DNA. By introducing the corresponding damping and noise terms [24,25], the equations are as follows:

(6) $$\begin{array}{ll} \frac{d\sigma_{z}}{\mathrm{dt}} & =-\Gamma_{1}(\sigma_{z}+1)+i\Omega_{p}(\sigma_{+}-\sigma_{-})\\ & \;+\frac{\mathrm{i\mu}E_{s}\text{exp}(-\mathrm{i\delta t})}{\hbar }\sigma_{+}-\frac{\mathrm{i\mu}E_{s}^{*}\text{exp}(\mathrm{i\delta t})}{\hbar }\sigma_{-}, \end{array}$$

(7) $$\begin{array}{lcrr} \frac{d\sigma_{-}}{\mathrm{dt}} & = & -(i\Delta_{p}+\mathrm{i\vartheta}+\Gamma_{2})\sigma_{-}-2i\Omega_{p}\sigma_{z} & \\ & & -\frac{2\mathrm{i\mu}E_{s}\text{exp}(-\mathrm{i\delta t})}{\hbar }\sigma_{z}+F_{n}, & \end{array}$$

(8) $$\begin{array}{lcrr} \frac{d^{2}\vartheta }{dt^{2}}+\frac{\mathrm{d\vartheta}}{\tau_{D}\mathrm{dt}}+\omega_{D}^{2}\vartheta =-\lambda \omega_{D}^{2}\sigma_{z}+\xi_{n}, & & & \end{array}$$

where $\lambda =\overset{n}{\underset{j=1}{\sum }}\frac{\hbar \kappa_{j}^{2}}{m_{j}\omega_{D}^{2}}$ is the coupling strength of DNA molecules and quantum dot. *ω*_D_ is the frequency of DNA longitudinal vibrational modes. The *δ*-correlated Langevin noise operator *F*_*n*_ represents the coupling between *ϑ* and *σ*_−_, the main cause of the decay of vibration mode. *F*_*n*_has zero mean value < *F*_*n*_ > = 0 and the correlation relation $<F_{n}(t)F_{n}^{+}(t^{\prime })>∼\delta (t-t^{\prime })$. The operator *ξ*_*n*_stands for the Brownian stochastic force, since the thermal bath of Brownian and non-Markovian processes will affect the vibration mode of DNA molecules [24,26]. The quantum effects on the DNA are only observed in the case *ω*_D_*τ*_D_ > > 1. The Brownian noise operator can be modeled as Markovian with the decay rate 1/_*τ*D_ of the vibration mode. Therefore, the Brownian stochastic force has zero mean value < *ξ*_*n*_ > = 0 and can be expressed as [26]

(9) $$\begin{array}{lcrr} <\;\xi^{+}(t)\xi (t^{\prime })\;>\;=\;\frac{1}{\tau_{D}\omega_{D}}\int \;\frac{1\;+\;\coth(\frac{\hbar \omega }{2k_{B}T})}{2\Pi }\omega e^{-\mathrm{i\omega}(t-t^{\prime })}\mathrm{d\omega .} & & & \end{array}$$

With the standard methods of quantum optics, the steady-state solution of Equations 6, 7, and 8 are expressed as follows when setting all the time derivatives to zero:

(10) $$\begin{array}{lcrr} \sigma_{0}=\frac{2i\Omega_{p}\sigma_{0z}}{\mathrm{i\lambda}\sigma_{0z}-\Gamma_{2}-i\Delta_{p}},\;\vartheta_{0}=-\lambda \sigma_{0z}, & & & \end{array}$$

where *σ*_0*z*_ is determined by Equation 15. To extend this formalism beyond weak coupling, we can always rewrite each Heisenberg operator as the sum of its steady-state mean value and a small fluctuation with zero mean value as follows: *σ*_−_ = *σ*_0_ + *δσ*_−_, *σ*_*z*_ = *σ*_0*z*_ + *δσ*_*z*_, and *ϑ* = *ϑ*_0_ + *δϑ*, which should be substituted into Equations 6, 7, and 8. We can neglect the nonlinear term *δϑδσ*_−_ safely. Since the optical drives are weak and classical, we will identify all the operators with their expectation values and omit the quantum and thermal noise terms [9]. Then the linearized Langevin equations can be written as follows:

(11) $$\begin{array}{lcrr} <\dot{\delta \sigma_{z}}> & = & i\Omega_{p}(<\delta \sigma_{-}^{*}>-<\delta \sigma_{-}>)-\Gamma_{1}<\delta \sigma_{z}> & \\ & & +\frac{\mathrm{i\mu}E_{s}\text{exp}(-\mathrm{i\delta t})}{\hbar }<\delta \sigma_{-}^{*}> & \\ & & -\frac{\mathrm{i\mu}E_{s}^{*}\text{exp}(\mathrm{i\delta t})}{\hbar }<\delta \sigma_{-}>, & \end{array}$$

(12) $$\begin{array}{lcrr} <\dot{\delta \sigma_{-}}> & = & -(i\Delta_{p}+\Gamma_{2})<\delta \sigma_{-}>-2i\Omega_{p}<\delta \sigma_{z}> & \\ & & -i(\vartheta_{0}<\delta \sigma_{-}>+<\delta \vartheta >\sigma_{0}) & \\ & & -\frac{2\mathrm{i\mu}E_{s}\text{exp}(-\mathrm{i\delta t})}{\hbar }<\delta \sigma_{z}>, & \end{array}$$

(13) $$\begin{array}{ll} <\ddot{\delta \vartheta }>+\frac{<\dot{\delta \vartheta }>}{\tau_{D}}+\omega_{D}^{2}<\delta \vartheta >=-\lambda \omega_{D}^{2}<\delta \sigma_{z}>. & \; \end{array}$$

From the approximations $<\delta \sigma_{z}>=\sigma_{z}^{+}\text{exp}(-\mathrm{i\delta t})+\sigma_{z}^{-}\text{exp}(\mathrm{i\delta t})$, $<\delta \sigma_{-}>=\sigma^{+}\text{exp}(-\mathrm{i\delta t})+\sigma^{-}\text{exp}(\mathrm{i\delta t})$ and < *δϑ* > = *ϑ*^ + ^exp(−*iδt*) + ^*ϑ*−^exp(*iδt*) [27], we can obtain:

(14) $$\begin{array}{lcrr} & & (-\Gamma_{1}\lambda^{2})\sigma_{0z}^{3}\;+\;(\frac{-\Gamma_{1}\lambda^{2}}{2}\;+\;2\Gamma_{1}\lambda \Delta_{p})\sigma_{0z}^{2}\;+\;(-\Gamma_{1}\Delta_{p}^{2}\;-\;\Gamma_{1}\Gamma_{2}^{2} & \\ & & \;\;\;\;\;+\Gamma_{1}\lambda \Delta_{p}\;-\;4\Gamma_{2}\Omega_{p}^{2})\sigma_{0z}\;=\;\frac{1}{2}\Gamma_{1}\Gamma_{2}^{2}+\frac{1}{2}\Gamma_{1}\Delta_{p}^{2}. & \end{array}$$

Now we get *σ*_0*z*_. Then *σ*_0_and *ϑ*_0_ are also known. All of the equations can then be solved completely. We finally obtain the part we are interested in, the equation:

(15) $$\begin{array}{lcrr} \sigma^{-}=Z(\delta )\sigma_{z}^{-}, & & & \end{array}$$

where the equations used are $G(\delta )=(\lambda \omega_{D}^{2})/(\delta^{2}+\mathrm{i\delta}/\tau_{D}-\omega_{D}^{2})$, $Z(\delta )=(\sigma_{0}G^{*}+2\Omega_{p})/(i\Gamma_{2}-\delta -\Delta_{p}-\vartheta_{0})$, *F*(*δ*) = (*σ*_0_*G* + 2*Ω*_*p*_)/(*iΓ*_2_ + *δ* − *Δ*_*p*_ − *ϑ*_0_), $\sigma_{0}=\frac{-2i\Omega_{p}\sigma_{0z}}{\Gamma_{2}+i\Delta_{p}-\mathrm{i\lambda}\sigma_{0z}}$, *ϑ*_0_ = −*λσ*_0*z*_ and finally

(16) $$\begin{array}{ll} \;\;\sigma_{z}^{+}\;=\;\frac{\mathrm{i\mu}E_{s}\sigma_{0}^{*}(i\Gamma_{2}+\delta -\Delta_{p}-\vartheta_{0})-2\mathrm{i\mu}E_{s}\Omega_{p}\sigma_{0z}}{\hbar (\mathrm{iG}\Omega_{p}\;-\;iZ^{*}\Omega_{p}\;+\;\Gamma_{1}\;-\;\mathrm{i\delta})(i\Gamma_{2}\;+\;\delta -\Delta_{p}\;-\;\vartheta_{0})}. & \;\\ & \; \end{array}$$

We can use the equations above and $\sigma_{z}^{-}=\sigma_{z}^{+*}$ to obtain the nonlinear optical susceptibility:

(17) $$\begin{array}{lcrr} \chi {\left(\omega_{s}\right)}_{\text{eff}}^{\left(3\right)}=\frac{N\mu^{3}\sigma^{-}}{3\varepsilon_{0}\hbar^{2}\Omega_{p}^{2}E_{s}^{*}}=\sum_{m}\chi^{3}(\omega_{s}), & & & \end{array}$$

where *N* is the number density of DNA-QDs and $\sum_{m}=\frac{N\mu^{4}}{3\varepsilon_{0}\hbar^{3}\Gamma_{2}^{3}}$.

## Results and discussion

To show the numerical results, we choose the realistic quantum dot-DNA system, in which a peptide quantum dot is coupled to several DNA molecules as illustrated in Figure 1. Although the DNA molecules in solution form can be distorted in mess, one can extend these molecules into linear form with electromagnetic field or fluid force [28]. In addition, the longitudinal vibrational frequency can be affected by the length of DNA molecules, which could just be considered as a factor affecting vibration frequency. In the theoretical calculation, we choose *ω*_D_ = 40 GHz and *τ*_D_ = 5 ns as the vibration frequency and lifetime of DNA molecules [22,29-31]. For our study, we can safely select the decay rate of the peptide quantum dot as *Γ*_1_ = 16 GHz for any practical purpose [32].

**Figure 2:**
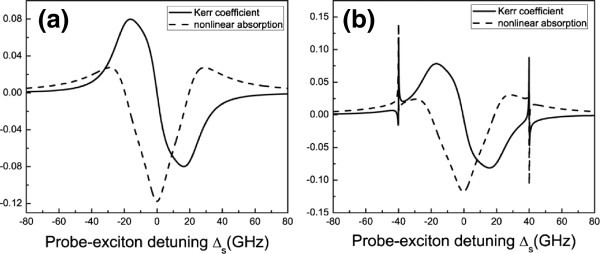
**The optical Kerr coefficient of probe beam (in units of***∑*_***m***_**) with pump beam on-resonance. (a)** The optical Kerr coefficient and nonlinear absorption as functions of probe-exciton detuning *Δ*_*s*_ in the case *λ*=0. **(b)** The optical Kerr coefficient and nonlinear absorption as functions of probe-exciton detuning *Δ*_*s*_ in the case *λ* = 2 GHz.

Figure 2a plots the optical Kerr coefficient Reχeff(3) (solid curve) and nonlinear absorption Imχeff(3) (dash curve) as functions of probe-exciton detuning *Δ*_*s*_ = *ω*_s_ − *ω*_eg_ with *Δ*_*p*_ = 0 and *λ* = 0, while Figure 2b shows optical Kerr coefficient Reχeff(3) (solid curve) and nonlinear absorption Imχeff(3) (dash curve) as functions of probe-exciton detuning *Δ*_s_ = *ω*_s_ − *ω*_eg_with *Δ*_*p*_ = 0 but *λ* = 2 GHz. It demonstrates that if we fix the pump beam on-resonance with the exciton frequency and scan the probe beam, we can obtain the large enhanced optical Kerr effect at *ω*_s_ = *ω*_eg_ − *ω*_D_ and *ω*_s_ = *ω*_eg_ + *ω*_D_. The origin of this phenomenon is the quantum interference between the vibration mode of DNA molecules and the beat of the two optical fields via the exciton when probe-pump detuning *δ*is adjusted equal to the frequency of the vibration mode of DNA molecules. If we ignore the coupling, *λ* = 0, the enhancement of optical Kerr effect will disappear completely as has been demonstrated in Figure 2a. Therefore, the importance of the coupling between quantum dot and DNA molecules is obvious since the enhancement of optical Kerr effect could not occur in such a system when *λ* = 0. Furthermore, we can propose a scheme to measure the frequency of the vibration mode of DNA molecules by making use of the phenomenon above. From Figure 2b, we can clearly see that as the frequency of the vibration mode is *ω*_D_ = 40 GHz, the two sharp peaks at ±40 GHz just match the mode frequency. This means that if we first adjust pump beam properly and scan the probe frequency across the exciton frequency in the spectrum, we can easily obtain the accurate vibration frequency of DNA, which implies some future potential applications.

**Figure 3:**
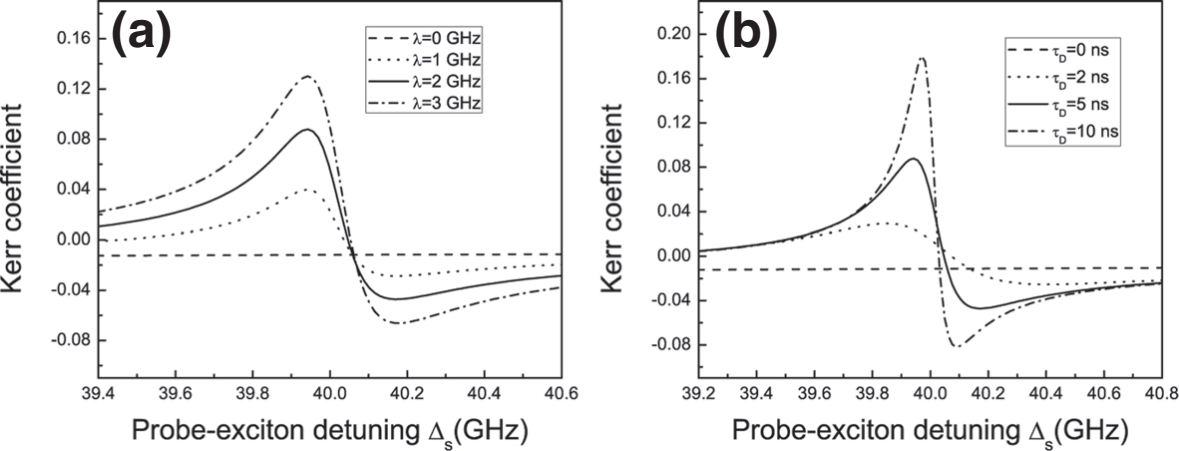
**Optical Kerr coefficient of probe beam with various coupling strengths and vibration lifetimes. (a)** The optical Kerr coefficient (in units of *∑*_*m*_) as functions of probe-exciton detuning *Δ*_*s*_ with pump beam on-resonance (*Δ*_*p*_ = 0) and different coupling strengths. **(b)** The optical Kerr coefficient (in units of *∑*_*m*_) as functions of probe-exciton detuning *Δ*_*s*_ with pump beam on-resonance (*Δ*_*p*_ = 0) and different vibration lifetimes.

**Figure 4:**
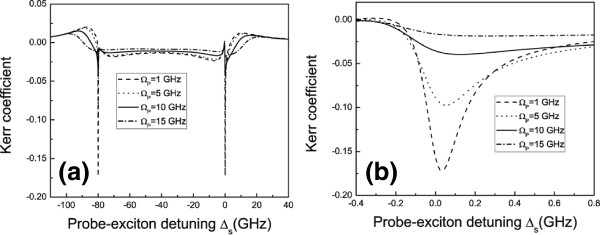
**Optical Kerr coefficient of probe beam with various Rabi frequencies of pump beam. (a)** The optical Kerr coefficient (in units of *∑*_*m*_) as functions of probe-exciton detuning *Δ*_*s*_ with pump beam off-resonance(*Δ*_*p*_ = *ω*_D_) and different Rabi frequencies. **(b)** The detailed part of (a) showed the relation between optical Kerr coefficient and Rabi frequency>

To explore the phenomenon above more carefully, we show the optical Kerr coefficient Reχeff(3) as functions of probe-exciton detuning *Δ*_*s*_ = _*ω*s_−_*ω*eg_with _*Δp*_ = 0 and different coupling strengths and vibration lifetimes in Figure 3a,b respectively. In Figure 3a, we see that the larger the coupling strength is, the higher the optical Kerr coefficient peak will be. Figure 4a shows that the optical Kerr coefficient peak increases monotonously with vibration lifetime *τ*_D_. We should not feel surprised about these results. Since the optical Kerr coefficient peak is caused by the coupling between DNA molecules and quantum dot, the peak will become more and more obvious when the coupling makes stronger. These results demonstrate that the coupling plays a key role in such a coupled system.

Figure 4a presents optical Kerr effects as functions of *Δ*_*s*_ with *Δ*_*p*_ = *ω*_D_ and different Rabi frequencies of the pump field, whose detail is shown in Figure 4a. We first notice that the probe beam experiences different optical Kerr coefficients when appearing in the pump beams with different intensities. When we pay attention to the detail (shown in Figure 4a), we find that by increasing the intensity of the pump beam, the optical Kerr effect will be weakened significantly. Therefore, we can see that the magnitude of optical Kerr effect can be tuned by controlling the light intensity, implying a method for regulating the nonlinear optical features of DNAs via coupling to quantum dots.

## Conclusions

In conclusion, we have proposed a theoretical model for DNA-quantum dot hybrid system in the presence of a strong pump laser and a weak probe laser. The coupling leads to the great enhancement of probe beam Kerr coefficient at two off-resonant points, which may be of potential use in frequency measurement. Furthermore, the relation between the optical Kerr coefficient of the probe beam and intensity of the pump beam may be utilized to control the strength of optical nonlinearity of the system. We believe that such a phenomenon may lead to a more profound understanding of nonlinear optical properties of the hybrid quantum dot-DNA system. We expect our consequences can be checked experimentally in the near future.

## Competing interests

The authors declare that they have no competing interests.

## Authors’ contributions

YL finished the main work of this article, including deducing the formulas, plotting the figures, and drafting the manuscript. KDZ conceived of the idea, provided some useful suggestion, and participated in the coordination. Both authors read and approved the final manuscript.
